# Strains Colonizing Different Intestinal Sites within an Individual Are Derived from a Single Founder Population

**DOI:** 10.1128/mbio.03456-22

**Published:** 2023-01-31

**Authors:** Vadim Dubinsky, Iris Dotan, Uri Gophna

**Affiliations:** a Shmunis School of Biomedicine and Cancer Research, George S. Wise Faculty of Life Sciences, Tel-Aviv University, Tel Aviv, Israel; b Division of Gastroenterology, Rabin Medical Center, Petah-Tikva, Israel; c Sackler Faculty of Medicine, Tel-Aviv University, Tel Aviv, Israel; Institut Pasteur

**Keywords:** *Bacteroides fragilis*, *Escherichia coli*, *Ruminococcus gnavus*, evolution, gut microbiome, metagenomics

## Abstract

Metagenomics has improved our understanding of commensal bacteria that colonize human intestines yet relies almost exclusively on fecal samples. Thus, spatial information about the niche range of these gut microbes and the level of specialized adaptation that they undergo has been inaccessible to fecal metagenomic studies. Here, we leveraged metagenomic data obtained through colonoscopy aspirates from three intestinal sites of healthy adults, and reconstructed metagenome-assembled genomes of several common gut bacteria to address intestinal site-specific evolution. We show that the genomes of bacterial strains at specific intestinal sites are clearly distinct yet are interrelated and are derived from a single founder strain colonizing multiple sites. We also reveal that within those intestinal sites, purifying selection is the dominant evolutionary force acting on Escherichia coli genomes within human hosts. Importantly, no site-specific adaptations at the level of accessory genes were detected, implying that these commensals are well-adapted to several host microniches and can therefore colonize multiple intestinal sites with high efficiency. Nevertheless, bacterial *in situ* growth rates differ markedly across different sections of the intestine. Metagenomics of aspirate samples can reveal unique strain- and intestinal tissue-specific genomic information. Such information may be critical for understanding bacterial contribution to gastrointestinal diseases, which involve only a part of the intestine, as is often the case in inflammatory bowel disease.

## INTRODUCTION

Microbial composition differs along the human gastrointestinal tract (1–3). Inevitably, spatial information is lost in most of the microbiome studies that rely on fecal samples as a proxy for the entire gut microbiota but probably reflect mostly the colonic microbiome. Some studies address this by sampling mucosal biopsy specimens at specific sites such as the colon (4) or at multiple sites in the upper and lower gastrointestinal tract (5, 6). However, biopsy specimens are biased in favor of tissue-adherent bacteria and contain a high fraction of human DNA (6, 7), thus hindering the use of shotgun metagenomics that requires sufficient sequence coverage, often leading researchers to rely on 16S rRNA amplicon sequencing instead. An alternative approach is to collect luminal content by aspiration during endoscopy from specific sites (6–9). Luminal aspirates were shown to be more like mucosal biopsy specimens than to stool, in terms of bacterial composition (6, 7) and functions (6), yet also contain sufficient bacterial DNA to perform shotgun metagenomics. Thus, these aspirates allow metagenome-based study of the mucosa-associated microbiome and its biogeography in the gastrointestinal tract. Nevertheless, no studies of bacterial genomes assembled from metagenomic data of aspirates are currently available. Thus, information about strain-specific variation and adaptation in the gastrointestinal tract is missing. Importantly, such genomes can uniquely address questions such as what the niche range of bacterial species in the gut is, and do they show niche-specific adaptive evolution (i.e., are there specific strains especially adapted to a particular intestinal site, such as the terminal ileum).

We took advantage of lumen shotgun metagenomes (6, 10) (*n* = 66; Table S1A in the supplemental material) from three locations in the large and small intestines: terminal ileum (TI), cecum, and descending colon (DC), sampled from 21 healthy individuals. For the metagenomes that had sufficient sequencing coverage (mean sequencing depth = 0.8 Gbp; s.e.m. = 0.45 Gbp, minimum depth for inclusion = 0.15 Gbp), we reconstructed 91 high-quality metagenome-assembled genomes (MAGs) of three common human gut species, representing different phyla: Escherichia coli (16 individuals, 51 MAGs), Bacteroides vulgatus (9 individuals, 26 MAGs), and Ruminococcus gnavus (6 individuals, 14 MAGs) from TI, cecum, or DC sites (Table S1B). Additionally, we reconstructed E. coli MAGs from fecal samples of 6 subjects that had corresponding aspirate samples. These MAGs provided unique insights into microbial evolution within the human host.

10.1128/mbio.03456-22.6 TABLE S1 Metagenomes used in this study and assembled genomes statistics. Download Table S1, XLSX file, 0.03 MB. Copyright © 2023 Dubinsky et al. 2023 Dubinsky et al. https://creativecommons.org/licenses/by/4.0/ This content is distributed under the terms of the Creative Commons Attribution 4.0 International license.

## RESULTS AND DISCUSSION

### Phylogenetic analysis indicates single founder lineages that colonize multiple intestinal sites within an individual.

We first analyzed the reconstructed MAGs of the three species from each intestinal location in the individuals using pairwise average nucleotide identity (ANI) comparisons (Fig. S1 and S2). ANI values of up to 99.9 to 99.95% between strains within an individual, compared to as low as 95.68 to 97.14% between strains across different individuals were observed, suggesting a strain-level individual-specific signal. To confirm that each of our reconstructed MAGs consisted of a single strain, the percentage of polymorphic sites was calculated. A median of 1.57 single nucleotide polymorphisms (SNPs) per kb sequence in the genomes suggested little intrasample strain heterogeneity, as was previously suggested (11).

10.1128/mbio.03456-22.1 FIG S1 Pairwise similarities comparison of genomic average nucleotide identity (ANI) values of the reconstructed E. coli MAGs from the lumen metagenomes. Each MAG is labeled with the sample name followed by the subject number. The lower triangle matrix is plotted and MAGs from different gastrointestinal locations of the same individual are displayed on the right side of the triangle. ANI values ranged from 95.68% to 99.95%. Download FIG S1, PDF file, 0.9 MB. Copyright © 2023 Dubinsky et al. 2023 Dubinsky et al. https://creativecommons.org/licenses/by/4.0/ This content is distributed under the terms of the Creative Commons Attribution 4.0 International license.

10.1128/mbio.03456-22.2 FIG S2 Pairwise similarities comparison of genomic average nucleotide identity (ANI) values of the reconstructed MAGs of B. vulgatus (A) and *R. gnavus* (B) from the lumen metagenomes. Each MAG is labeled with the sample name followed by the subject number. The lower triangle matrix is plotted and MAGs from different intestinal locations of the same individual are displayed on the right side of the triangle. ANI values ranged from 97.14% to 99.92% in (A) and from 96.51% to 99.9% in (B). Download FIG S2, PDF file, 0.6 MB. Copyright © 2023 Dubinsky et al. 2023 Dubinsky et al. https://creativecommons.org/licenses/by/4.0/ This content is distributed under the terms of the Creative Commons Attribution 4.0 International license.

**FIG 1:**
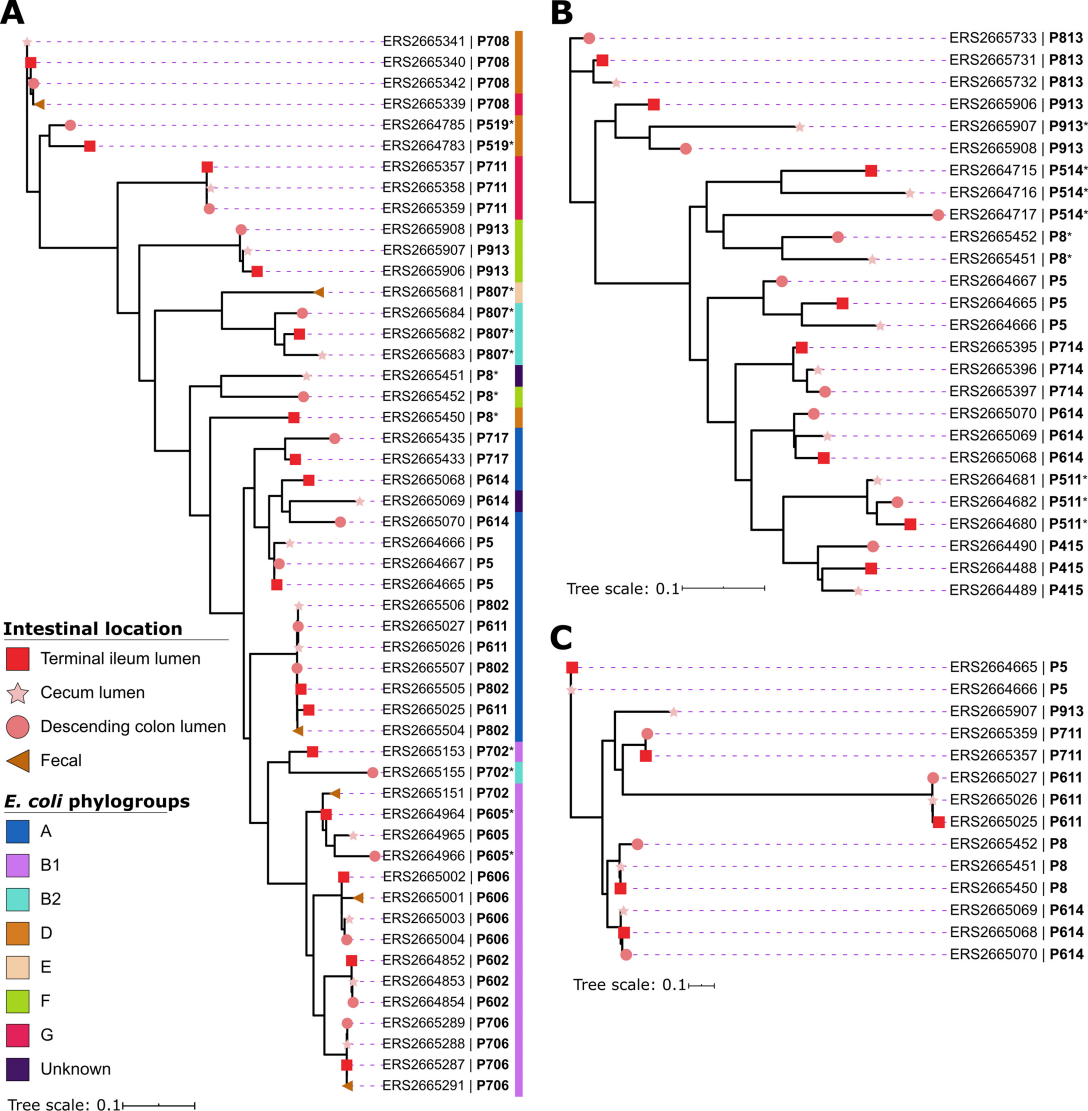
Phylogenetic structure of the reconstructed MAGs of three common species from the lumen metagenomes derived from three intestinal locations of healthy subjects. (A) Escherichia coli strains (51 MAGs) from 16 individuals. (B) Bacteroides vulgatus (26 MAGs) from 9 individuals. (C) Ruminococcus gnavus (14 MAGs) from 6 individuals. Each node is labeled with the sample name followed by the subject number. Intestinal locations are denoted with colored shapes next to each node. In panel A, there are, in addition, 6 E. coli MAGs reconstructed from fecal samples. The phylogroups of the E. coli strains are denoted with a colored bar next to the labels. The phylogenetic trees were reconstructed with RAxML (parameters “-p 1989 -m GTRCAT”) based on species-specific marker genes defined as core, that were present in ≥95% of the MAGs. The phylogenetic tree scale is nucleotide substitutions per site. Asterisks next to the subjects’ names represent MAGs with potential high within-strain variability (>5 SNPs per kb sequence).

Next, we investigated in higher resolution the phylogenetic structure of the reconstructed MAGs of our representative species. Overall, a strong strain-specific genomic signature within an individual’s intestinal locations was observed (Fig. 1A to C). In most subjects, strains from TI, cecum, and DC clustered together and apart from strains from other subjects, indicating a probable single founder strain for the three niches. Exceptions (unusual branching order or particularly long branches) were cases where there was relatively high-potential within-MAG strain variability (>5 SNPs per kb sequence [11]), which probably represent a mixture of strains within a site (marked by asterisks in Fig. 1). There was no consistent pattern of similarity between strains in intestinal locations within an individual (i.e., TI was not consistently more like the cecum than to the DC across subjects, etc.). We then attempted to reconstruct MAGs from fecal metagenomes available for some of the same individuals, and for E. coli, we had sufficient genome coverage to do so. Four out of the six E. coli MAGs we reconstructed from fecal metagenomes grouped with the corresponding subject’s intestinal samples. The fecal E. coli strains of subjects P606 and P706 were more like their corresponding DC sites, while in subjects P708 and P802, the fecal strains showed higher similarity to the TI based on the small amount of single nucleotide polymorphisms (SNPs) between them (Table S2). In contrast, the fecal strains of subjects P807 and P702 might be derived from sites not sampled for luminal content (6, 10), since they differed from the luminal strains of all three intestinal sites in those patients according to the SNP analysis (Table S2).

10.1128/mbio.03456-22.7 TABLE S2 SNPs between fecal and corresponding lumen MAGs. Download Table S2, XLSX file, 0.01 MB. Copyright © 2023 Dubinsky et al. 2023 Dubinsky et al. https://creativecommons.org/licenses/by/4.0/ This content is distributed under the terms of the Creative Commons Attribution 4.0 International license.

We also looked for minor E. coli strains that may be present in all the three different intestinal sites within individuals by identifying shared SNPs that differed from that individual major strain (the assembled MAG) and did not exceed 50% frequency in any site (to filter out the SNPs within the strains of the major lineage). In 12 of 13 individuals we could detect over 20 SNPs that met our quality criteria (see Materials and Methods) and were observed in all three intestinal sites (Table S3). This analysis indicates that nondominant E. coli lineages can also successfully stably colonize multiple sites within the human intestine.

10.1128/mbio.03456-22.8 TABLE S3 Number of SNPs shared across intestinal sites of individuals that indicate the presence of minor lineages of E. coli. Download Table S3, XLSX file, 0.01 MB. Copyright © 2023 Dubinsky et al. 2023 Dubinsky et al. https://creativecommons.org/licenses/by/4.0/ This content is distributed under the terms of the Creative Commons Attribution 4.0 International license.

Finally, an analysis of E. coli phylogroups (Fig. 1A) revealed that our strains originated from a diverse array of lineages (A, B1, B2, D, E, F, G), indicating that many E. coli lineages can successfully colonize healthy humans.

**FIG 2:**
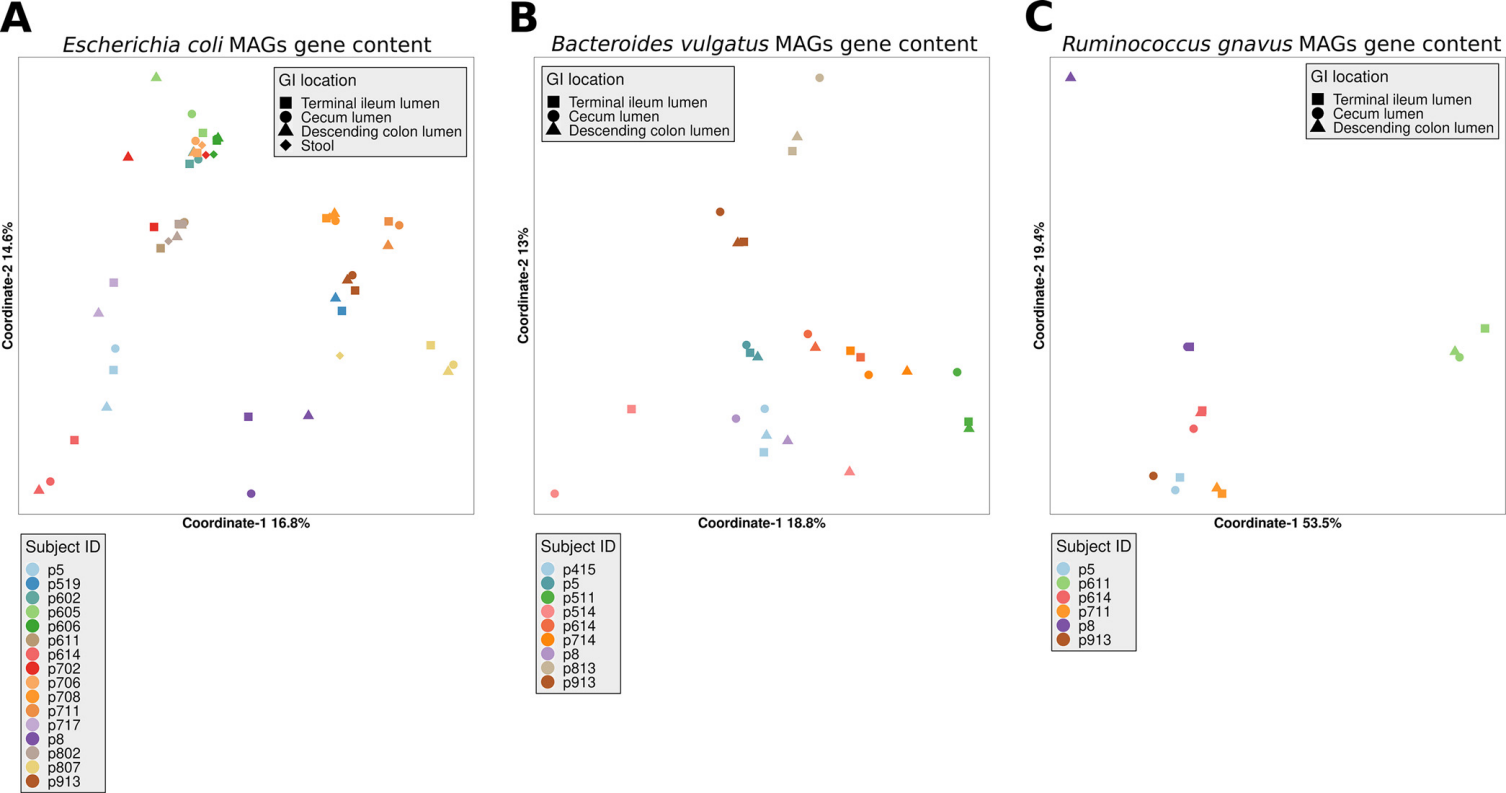
Gene content analysis of each species’ MAGs in different individuals and intestinal locations, plotted with principal coordinates analysis (PCoA) according to Jaccard distance (overall dissimilarity of the presence or absence of genes). Functional profiles were generated with EggNOG mapper based on KEGG Orthology. PCoA of E. coli (A), B. vulgatus (B), and *R. gnavus* (C) is shown. The shapes denote different intestinal locations, and all the corresponding samples of each subject are colored accordingly.

We functionally annotated the genomes of the three species (based on KEGG Orthology) and analyzed the similarity in the gene content (Fig. 2) between the strains. Similar to the phylogeny and ANI analyses, each species’ MAGs from different intestinal locations within an individual tended to cluster together, although some strains from different subjects were almost indistinguishable from one another in terms of gene content, and formed tight clusters, thus probably sharing most genes. To confirm that strains from three intestinal sites within individuals shared most of their genes, we looked for gene gain/loss events in a subset of high-completeness E. coli MAGs (*n* = 27) from 9 subjects. By mapping all the metagenomic reads from one site against the assembled contigs from a MAG of another site in an individual, and analyzing read coverage at coding genes, we could not detect either missing or acquired genes between the intestinal sites within an individual. In contrast (and expectedly), by applying this method to reads/contigs across individuals, we found that about 300 to 400 genes were missing between distantly related strains, and 10 to 30 genes differed between closely related strains (defined as such based on the phylogenetic tree in Fig. 1A). These genes included both metabolic genes and genes typical to mobile genetic elements (prophages and transposable elements). Thus, intersubject variation explained most of the dissimilarity in gene content between the MAGs of each species (PERMANOVA: E. coli, *R*^2^ = 0.76, *P* < 0.001; B. vulgatus, *R*^2^ = 0.67, *P* < 0.001; *R. gnavus*, *R*^2^ = 0.87, *P* < 0.001). Similarly, all the recovered plasmids in the respective metagenomes of these 27 E. coli strains that could be reliably assigned to E. coli were shared across all intestinal locations in these individuals (Table S4). Taken together, core-gene phylogeny and gene content data are compatible with a scenario in which strains occupying different intestinal sites within an individual are derived from a single founder strain, which has later evolved independently at the three intestinal sites (sympatric diversification). Importantly, no site-specific adaptation at the level of accessory (noncore) gene content, including the ones found on plasmids, could be detected.

10.1128/mbio.03456-22.9 TABLE S4 Assembled *Enterobacteriaceae* plasmids annotation and mapping. Download Table S4, XLSX file, 0.03 MB. Copyright © 2023 Dubinsky et al. 2023 Dubinsky et al. https://creativecommons.org/licenses/by/4.0/ This content is distributed under the terms of the Creative Commons Attribution 4.0 International license.

### *In situ* bacterial growth rates within individuals.

**FIG 3:**
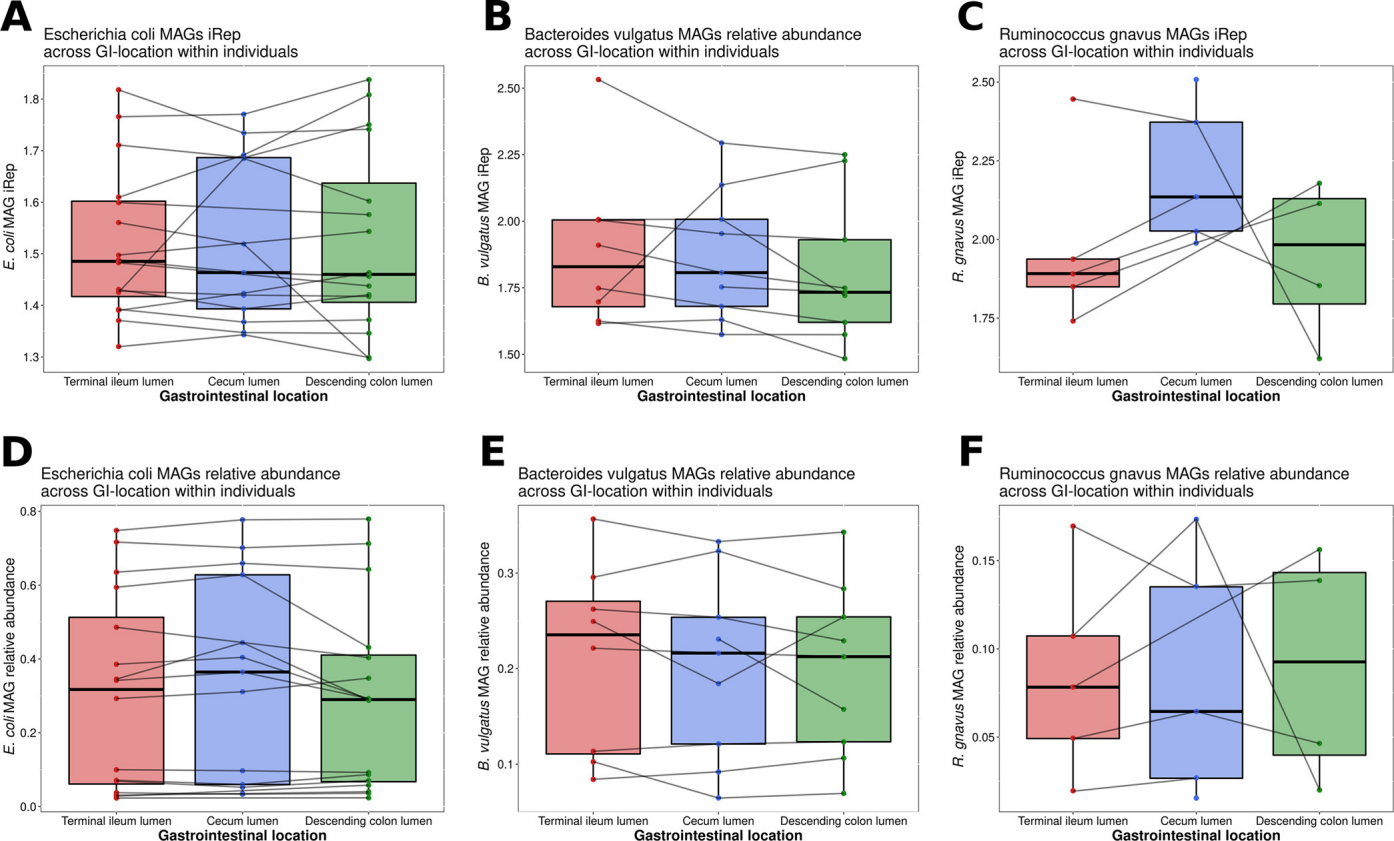
Inferred growth rates and relative abundance of the reconstructed MAGs of three common species from the lumen metagenomes across three intestinal locations of healthy subjects. (A, B, C) iRep (genome replication index) values across the terminal ileum, cecum, and descending colon for the genomes of E. coli (A), B. vulgatus (B), and *R. gnavus* (C). (D, E, F) Relative genome abundance of each strain of E. coli (D), B. vulgatus (E), and *R. gnavus* (F). MAGs from the intestinal locations of a specific individual are connected with a line crossing the boxplots.

Next, we analyzed the growth dynamics of the strains within the intestinal tract of the subjects (Fig. 3), based on the principle that cells that actively replicate their genomes have higher coverage near the origin of replication, compared to terminus (12). We used iRep (genome replication index [13]) to infer the growth rate of the reconstructed MAGs, which measures the activity of the species at the time of sampling and hence represents its physiological state. Surprisingly, E. coli strains within individuals exhibited overall similar growth rates in the small and large intestines (Fig. 3A), although in several cases the growth rate changed from one intestinal location to another. For B. vulgatus strains, besides one exception, growth rates were somewhat lower in the DC (Fig. 3B), while *R. gnavus* grew faster in the cecum compared with the other sites (Fig. 3C). These relatively similar replication rates across TI, cecum, and DC, are somewhat surprising given the differences in the levels of available nutrients and various host factors (pH, oxygen, and antimicrobial peptides [3]), and imply that these successful intestinal symbionts, and especially E. coli, are well-adapted to all three sites. The relative abundances of each species’ MAGs were even more stable than the corresponding iRep values (Fig. 3D to F) across intestinal sites. *R. gnavus* and B. vulgatus had a significantly higher replication rate than E. coli (Fig. S3B; Kruskal-Wallis, *P* < 0.001): *R. gnavus* had a median iRep of 2.01, while B. vulgatus and E. coli had a median iRep of 1.75 and 1.46, respectively. A comparable replication rate for E. coli from infant gut (about 1.3) was previously reported (14). However, the median relative abundance of *R. gnavus* (7.1%) was substantially lower than that of B. vulgatus and E. coli (21.9% and 29.2%, respectively; Fig. S3A; Kruskal-Wallis, *P* < 0.01). High bacterial growth rate was not associated with high bacterial abundance, but rather a negative trend was detected (Fig. S3C; spearman *r* = –0.258, *P* = 0.0134). A possible explanation for this observation is that at the time of sampling, E. coli and B. vulgatus may be more adherent, while *R. gnavus* is shed more in the feces and must replenish its number through faster growth to avoid washout. Lastly, despite having highly similar genomes, E. coli strains from the intestines had different replication rates (Fig. S4A) and relative abundance (Fig. S4B) than strains from the corresponding fecal samples.

10.1128/mbio.03456-22.3 FIG S3 Inferred growth rate compared to relative abundance of the reconstructed MAGs of E. coli, B. vulgatus, and *R. gnavus*, aggregated for all intestinal locations. (A) Relative genome abundance of each species. (B) iRep (genome replication index) values of the genome of each species. (C) High iRep is not associated with high genome abundance, but rather a negative correlation is observed (spearman *r* = −0.258, *P* = 0.0134). Download FIG S3, PDF file, 0.4 MB. Copyright © 2023 Dubinsky et al. 2023 Dubinsky et al. https://creativecommons.org/licenses/by/4.0/ This content is distributed under the terms of the Creative Commons Attribution 4.0 International license.

10.1128/mbio.03456-22.4 FIG S4 Inferred growth rate and relative abundance of E. coli strains from six subjects that had fecal samples corresponding to the lumen aspirates. iRep values (A) and relative genome abundance (B) for the MAGs obtained from the terminal ileum, cecum, descending colon, and fecal metagenome samples. MAGs from the samples of each individual are connected with a line crossing the boxplots. Download FIG S4, PDF file, 0.2 MB. Copyright © 2023 Dubinsky et al. 2023 Dubinsky et al. https://creativecommons.org/licenses/by/4.0/ This content is distributed under the terms of the Creative Commons Attribution 4.0 International license.

### Effects of antibiotic treatment on E. coli within individuals.

Out of the 16 individuals from whom we reconstructed E. coli genomes, 12 were treated with the antibiotic ciprofloxacin (Table S1A), and their lumen aspirates were sampled either immediately after antibiotic treatment termination (*n* = 8) or following 21 days of recovery (*n* = 4). We then focused on E. coli, because ciprofloxacin resistance is well understood in this species and resistance mutations have been well characterized. We thus extracted the primary target genes for ciprofloxacin, DNA gyrase A (*gyrA*), and topoisomerase C (*parC*) from the E. coli MAGs and checked for point mutations in the three positions in *gyrA*/*parC* known to confer resistance (15). Nineteen of 27 E. coli MAGs obtained from individuals sampled immediately after antibiotic treatment had three resistance mutations known to confer complete resistance to ciprofloxacin. In contrast, 3 of 11 MAGs obtained after 21 days of recovery had these three resistance mutations while the rest had either one or no mutations. Of the 10 genomes from the nonantibiotics-treated individuals, five had only one mutation, and the remaining five had no mutations (Table S1B).

Antibiotic treatment could confound population genetics analysis if sensitive strains are killed off by the drug, creating a population bottleneck, and new strains are introduced, for example by invasion of resistant bacteria from outside. For 27 high-quality and completeness E. coli MAGS from 9 individuals that did not have multiple lineages (<5 SNPs/Kb within-MAG), we compared total unique mutations per GI site per individual. We observed comparable numbers of unique mutations regardless of antibiotic treatment or numbers of resistance mutations (Kruskal-Wallis, *P* > 0.05; Fig. S5A; Table S1B). Lastly, resistant E. coli strains observed immediately after antibiotic treatment had reduced replication rates (Kruskal-Wallis, *P* < 0.05; Fig. S5B; Table S1B). Overall, although we detected antibiotic-resistant strains, especially from samples collected immediately following treatment, we also identified partially resistant (15) or antibiotic-sensitive strains in our MAGs set. Thus, the within-individual population structure we observed can be generalized, regardless of the resistance status of the strains in questions.

10.1128/mbio.03456-22.5 FIG S5 Effects of antibiotic treatment on total unique mutations and on inferred growth rate in E. coli MAGs. The samples from the subjects were divided into three groups: those obtained immediately after antibiotic termination, after 21 days of recovery from antibiotic, and without antibiotic treatment. (A) Total unique mutations per GI site per individual, no significant difference was observed (Kruskal-Wallis, *P* > 0.05). (B) Inferred growth rate, a star denotes significant difference between “no-Abx” versus “immediately after Abx” groups. Download FIG S5, PDF file, 0.3 MB. Copyright © 2023 Dubinsky et al. 2023 Dubinsky et al. https://creativecommons.org/licenses/by/4.0/ This content is distributed under the terms of the Creative Commons Attribution 4.0 International license.

### Purifying selection dominates the genomes of E. coli strains within individuals.

**FIG 4:**
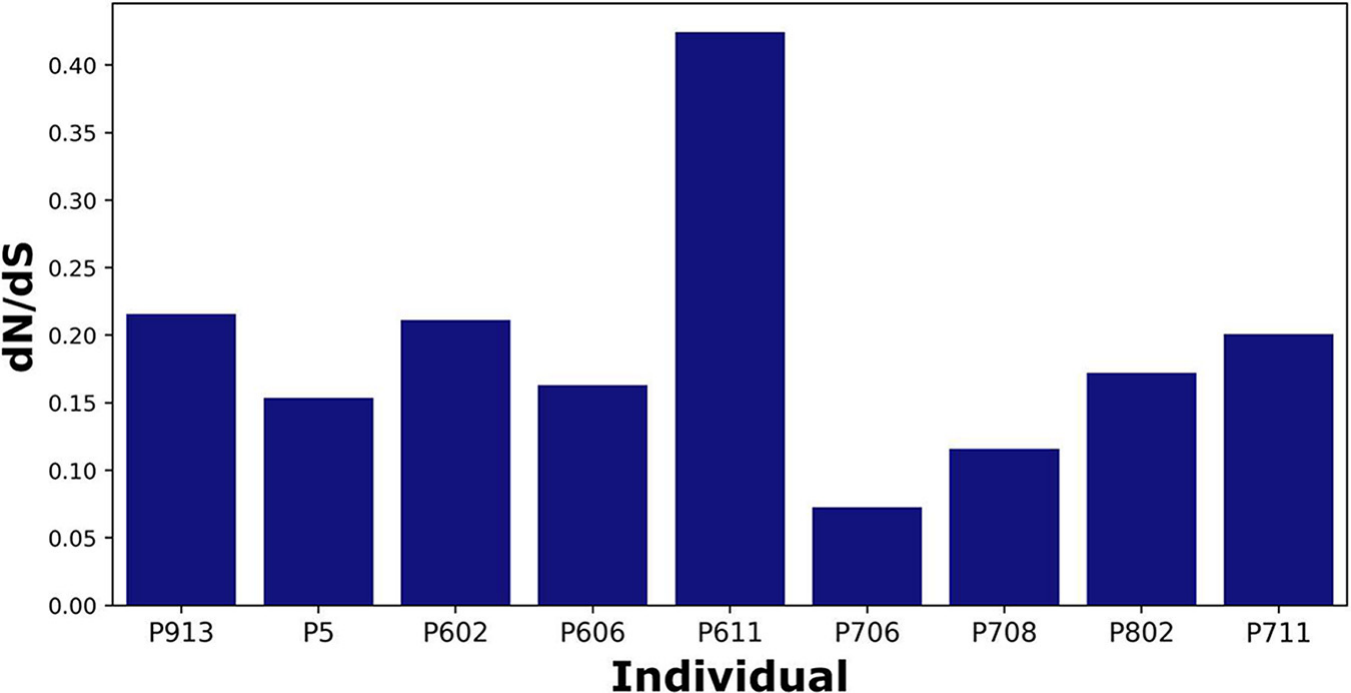
Mutation analysis in a subset of high-quality E. coli MAGs (*n* = 27) from 9 individuals. The ratio of nonsynonymous to synonymous polymorphisms in protein-coding genes (dN/dS) of the E. coli strains within individuals.

Previous studies based on bacterial isolate genomes have shown that most gut commensals are subject to strong purifying selection within the host, but also experience rather frequent adaptive evolution (16, 17). In contrast, E. coli was shown to evolve under a mostly neutral regime within its human host, presumably due to a small effective population size (18). To examine the selection regime at different intestinal sites, we identified mutations in the 27 E. coli strains from 9 subjects described above and estimated the ratio of nonsynonymous versus synonymous polymorphisms (Table S5), dN/dS, a widely used measure of natural selection operating on protein coding genes (19). dN/dS values of around 1 imply neutral evolution, while dN/dS < 1 can be interpreted as purifying selection, and dN/dS > 1 as positive selection. The advantage of studying within-host evolution using MAGs instead of isolates is that one can observe and quantify mutations that have not yet reached fixation—positions that are polymorphic within a sympatric population of cells—increasing the sensitivity of analysis. The dN/dS ratios within individuals (Fig. 4) ranged from 0.073 to 0.424 (mean ± s.e.m.: 0.1922 ± 0.0328), indicating that purifying selection operates on E. coli in the intestines of all individuals studied, as can be expected for a large population of bacteria in the highly competitive environment of the gut, and in line with previous studies of other common gut commensals (16, 20).

10.1128/mbio.03456-22.10 TABLE S5 Synonymous, nonsynonymous, and frameshift mutations summary in E. coli strains from the intestinal sites. Download Table S5, XLSX file, 0.5 MB. Copyright © 2023 Dubinsky et al. 2023 Dubinsky et al. https://creativecommons.org/licenses/by/4.0/ This content is distributed under the terms of the Creative Commons Attribution 4.0 International license.

To test whether some mutations were adaptive for a specific intestinal site, we analyzed mutations unique to a site and likely to be on their way to fixation (if the fraction of reads showing the variant allele was ≥80% among all reads covering a particular position). When surveying such fixed and near-fixed mutations (either unique to one intestinal site or common to any two sites; Table S5A and B), a large fraction of these mutations occurred at intergenic regions, which, in bacteria, are more likely to be neutral (Table S5A). For nonsynonymous mutations at coding genes, we observed no gene with such mutations that was encountered multiple times across subjects (Table S5C), implying that none of these alleles were adaptive enough to be fixed independently in multiple cases. Thus, it is likely that the mutational variants we observed were either neutral or slightly deleterious. By comparing the overall number of fixed and near-fixed mutations (both synonymous and nonsynonymous; Table S5A) to that previously observed in isolates from human feces (about 7 × 10^−7^ mutations per base per year, and thus fewer than 4 per year for a typical E. coli genome [18]), and assuming a similar rate of mutation, we could infer that the E. coli genomes we reconstructed have already been diverging at their respective intestinal sites for approximately 5.5 years. It is important to note that these aspirate-derived E. coli strains come from a relatively large cell population, and their relative abundance (median of 29.2%, Fig. S3A) was much higher than what is typically observed for E. coli in fecal metagenomes from healthy individuals (16, 20). Thus, these bacteria are highly likely to exist under conditions that exert strong purifying selection, where less fit mutants are likely to become extinct.

Taken together, these analyses indicate that purifying selection is dominant in E. coli evolution within hosts, and that the ancestral colonizing strains were adequately preadapted to all three intestinal sites, since no site-specific adaptive signature could be detected. This contrasts with the rapid adaptation observed in experiments where mice were colonized with E. coli of human origin (17), which originally were probably poorly adapted to a murine host.

Previous studies based on fecal metagenomes have reported intrasubject stability in the overall microbiota composition over the course of a year (21). Dominant subject-specific strain variants have been observed over time in multiple species in the human microbiome (22). Notably, the same bacterial strains were even shown to colonize multiple human organs (oral and skin) simultaneously in premature infants (14) and in fecal and vaginal samples from mothers during pregnancy (23). Our findings based on gut lumen metagenomes support and extend those observations. We show that subject-specific strains of intestinal bacteria are found across different sections of the intestine. This contrasts with the gut site specificity of different strains that was observed for several fish commensals (24). The dissimilarity between these studies could stem from the ecological differences between the fish midgut and hindgut that may be much larger than differences between the human terminal ileum and colon. Accordingly, it remains to be determined whether such “generalist” strains can also inhabit additional gastrointestinal sites (e.g., duodenum and stomach) or diseases-affected tissues, as in the case of inflammatory bowel disease (25), where inflammation can be restricted to a single intestinal section. Altogether, our study demonstrates that genome-resolved methods applied to lumen metagenomes enable the determination of the niche range of bacterial strains and the *in situ* selection regimes within the human host. Our analysis was limited to a small number of MAGs from a few individuals and reconstructed from only three abundant bacterial species. Future studies should collect luminal aspirates from larger cohorts (including patients with gastrointestinal diseases) as well as from additional intestinal sites and multiple time points. This will enable more detailed insights into microbial activity and evolution along the entire gastrointestinal tract, the modifying factors, and how it affects human health and disease.

## MATERIALS AND METHODS

### Pipeline for the assembly and analysis of genomes from metagenomes.

To assemble metagenome-assembled genomes (MAGs) of the three bacterial species, we applied the following bioinformatic pipeline to each metagenome: (i) Human-derived reads were removed by mapping them against the human genome (GRCh38) with Bowtie2 v2.2.9 (26). (ii) The remaining reads were mapped against a reference database of each species’ pangenome (separately) to retain the closely matching reads using MIDAS (27). (iii) Samples with a sufficient amount of mapped reads (>300,000) were assembled with metaSPAdes v3.14 (28) with the “–meta” option, and, including the read corrector BayesHammer. The resulting scaffolds were binned with MetaBAT2 (29) to obtain species-level genome bins. (iv) Completeness and contamination measures of the MAGs were assessed using CheckM (30) with a taxonomy-specific workflow. MAGs with completeness <75% and contamination >5% were discarded. In an attempt to obtain more complete and less contaminated MAGs, instead of binning in step iii, contigs were screened with BLASTN against the NR database, and only those that matched the specific species of interest at ≥95% identity were retained. The amount of strain-level heterogeneity was estimated with CMSeq (https://github.com/SegataLab/cmseq), which calculates the polymorphism at each position in the contigs (a position was considered nonpolymorphic if the dominant allele frequency was >80%). (v) The relative abundance of each MAG in the metagenomes was calculated by mapping reads against the reconstructed MAG from the same sample using Bowtie2 and to avoid counting reads from closely related strains, reads with an edit distance (mismatches) of ≥ 2 bases to the contigs were discarded. Genome replication rate for each MAG was calculated with the iRep algorithm (13). Average nucleotide identity (ANI) between each pair of MAGs was calculated with FastANI (31). Gene content analysis was done with EggNOG mapper (32) based on EggNOG orthology data. Phylogenetic structure of each species was built using PhyloPhlAn-3 (33), each phylogeny was based on a set of species-specific marker genes (E. coli - 2672, B. vulgatus - 2136, *R. gnavus* - 1788) identified using the UniRef90 database.

### Single nucleotide polymorphisms analysis between fecal and lumen genomes.

To compare the E. coli MAGs assembled from fecal samples (*n* = 6) to the corresponding subject’s (*n* = 6) lumen samples MAGs (*n* = 17), we performed single nucleotide polymorphisms (SNPs) analysis between each fecal and lumen genomes. The metagenomic reads that composed a MAG from a fecal sample were mapped against each corresponding intestinal (lumen) sites MAGs in each subject with Bowtie2. SNP positions between the fecal and lumen MAGs were detected with SAMtools v1.3.3 (34) function *mpileup* and VarScan v2.3.9 (35) function *mpileup2snp*. Only SNP positions with a minimum read coverage of 8 were included in the analysis. We counted both the total SNPs and SNPs with the fraction of reads mapping to the variant allele of ≥80% (dominant SNPs), between each fecal-lumen genomes pair.

### Evolution analysis and dN/dS calculation.

For a subset of E. coli MAGs (*n* = 27) with high completeness (mean of 97.2%) and low contamination (mean of 0.3%) from 9 subjects, we performed variant calling analysis using the PATRIC server (36) with default parameters, by mapping metagenomic reads that composed a MAG from each intestinal site against the MAG with the highest completeness in each subject. Variants were identified in coding and noncoding regions in the genome, and were classified as synonymous, nonsynonymous, and insertion/deletion. For dN/dS ratio calculation, we considered only single nucleotide polymorphisms (SNPs) in coding genes, with average reads coverage ≥10 and with the fraction of reads mapping to the variant allele ≥50%. For dN/dS analysis, the codons of each variant and reference alleles of all SNPs were concatenated into a quasi-coding sequence for each site per subject. We then used the Selecton server (37) with default settings to obtain dN/dS for the aligned coding sequences. For the analysis of fixed/near fixed mutations, we considered SNPs in both coding genes and intergenic regions, with the fraction of reads mapping to the variant allele ≥80%.

### Gene gain and loss events.

To detect gene gain and loss events in a subset of high-quality E. coli MAGs (*n* = 27) from 9 subjects, the assembled MAGs were annotated with Prokka v1.13.3 (38) to obtain coding genes annotations and position over the assembled contigs. For each subject, all the metagenomic reads from one site were mapped against the assembled contigs of a MAG from another site (all versus all) using Bowtie2. To obtain reads coverage at coding genes location, BEDtools v2.26.0 (39) function *multicov* was used, provided with mapping (.bam) and gene coordinates (.gff) files. Genes with fewer than 3 mapped reads in the target genome but ≥10 reads at the reference genome were considered missing.

### Plasmid assembly and analysis.

To assemble plasmids from the subset of samples with high-quality E. coli MAGs (*n* = 27), metaSPAdes with “–plasmid –meta” options were used on the metagenomic reads that were trimmed for quality and filtered for human reads as described above. The resulting assembled scaffolds were analyzed with BLASTN against PLSDB (a resource containing an extensive set of verified plasmid from NCBI with additional annotations; [40]). Plasmids with <80% similarity and <1,000 nucleotides alignment length to a database reference were discarded, and only plasmids matching to E. coli as the first or second-best hit were retained. To detect plasmid gain/loss events, we followed the same procedure with the contigs mapping as described above for chromosomal genes.

### Identification of ciprofloxacin-resistance mutations.

To identify mutations predicted to confer resistance to ciprofloxacin, the primary target genes for ciprofloxacin, DNA gyrase A (*gyrA*), and topoisomerase C (*parC*) were extracted from the E. coli reconstructed MAGs’ coding genes, which were predicted and annotated by Prokka v1.13.3 (38). Point mutations in these genes in positions known to confer resistance to ciprofloxacin were identified as described in (25).

### Minor strains identification and their tracking across multiple metagenomes.

To detect the presence of minor (nondominant) strains of E. coli across the lumen samples of individuals, we performed SNPs analysis that compared metagenomic reads from those samples and the assembled E. coli MAGs from the same individuals. Specifically, for each subject with metagenomes obtained from three intestinal sites (*n = *13), the MAG of highest completeness and lowest contamination level was selected and used as a reference. The metagenomic reads from each of the samples were mapped against the reference MAG with Bowtie2, and SNPs were called with SAMtools v1.3.3 function *mpileup* and VarScan v2.3.9 function *mpileup2snp*. Only SNP positions with a minimum read coverage of 8 and minimum variant allele frequency of 20% were analyzed. To discard SNPs that may originate from major strain lineages, SNPs of the variant allele at frequencies of >50% were filtered out. Identical SNPs, as defined to be on the same genomic contig, position, and nucleotide variant across all 3 lumen samples (intersection of the 3 SNPs tables per individual) were considered shared across sites. A minimum threshold of ≥20 shared SNPs per individual was considered to indicate the presence of the lineage with appropriate confidence.

### Data availability.

The assembled MAGs in this study were deposited in NCBI-WGS under the Bioproject accession number PRJNA647853. The shotgun metagenomic data used to assemble the MAGs were retrieved from NCBI SRA with Bioproject accession number PRJEB28097.
