# Supplementary figures and images for: Strains Colonizing Different Intestinal Sites within an Individual Are Derived from a Single Founder Population
Source: mBio. 2023 Jan 31;14(1):e03456-22. doi: 10.1128/mbio.03456-22 (PMC9972980; doi:10.1128/mbio.03456-22)

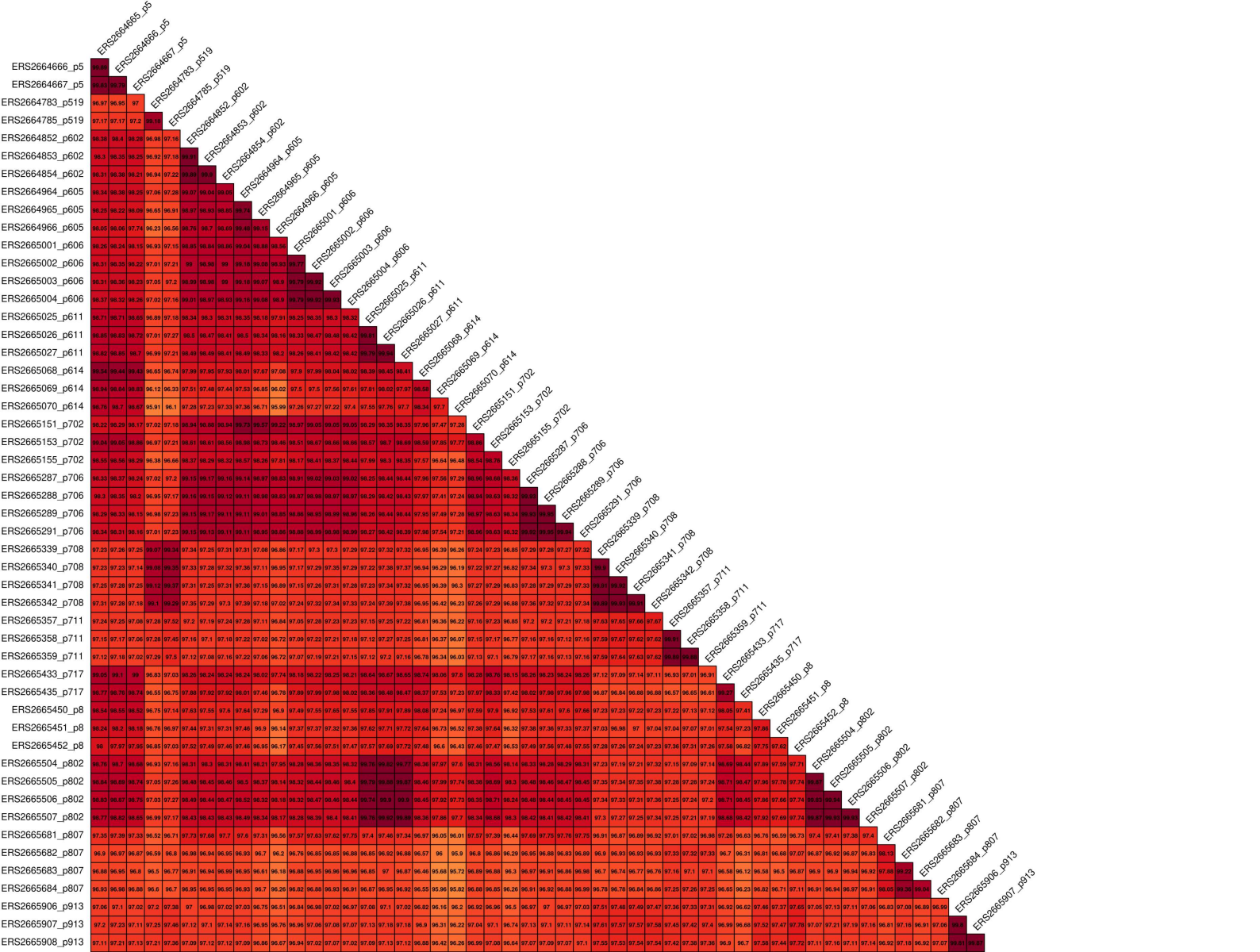

95.68 96.11 96.54 96.96 97.39 97.82 98.24 98.67 99.1 99.52 99.95

Supplement: FIG S1 [file mbio.03456-22-s0001.pdf]

A

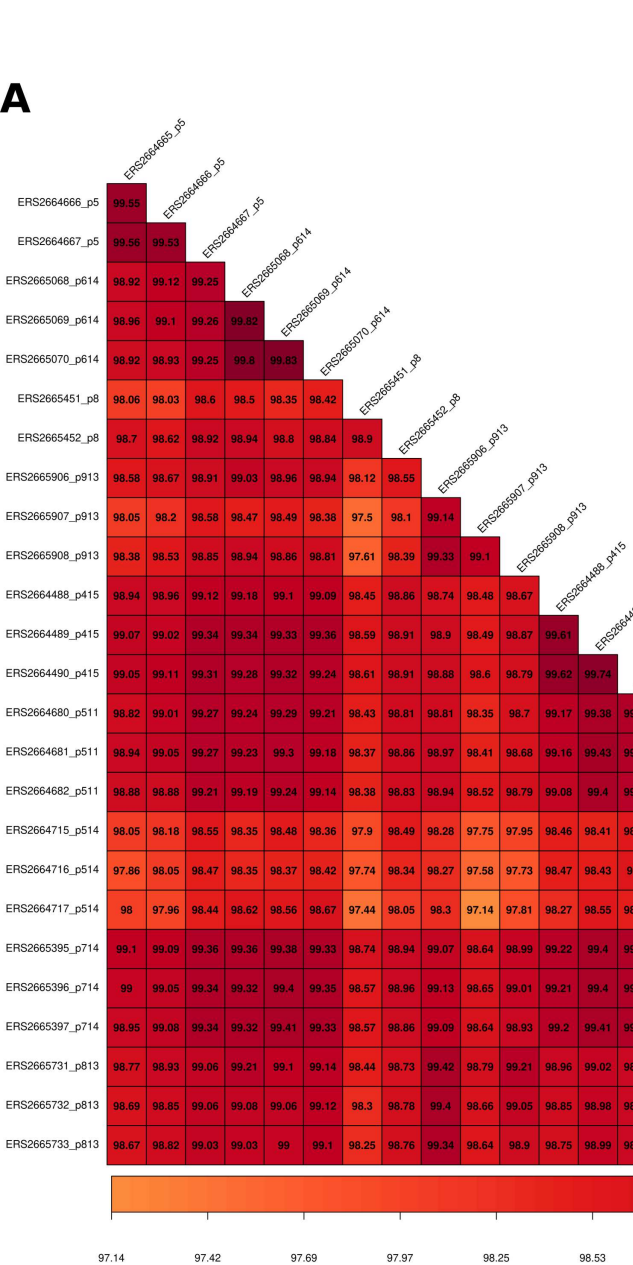

B

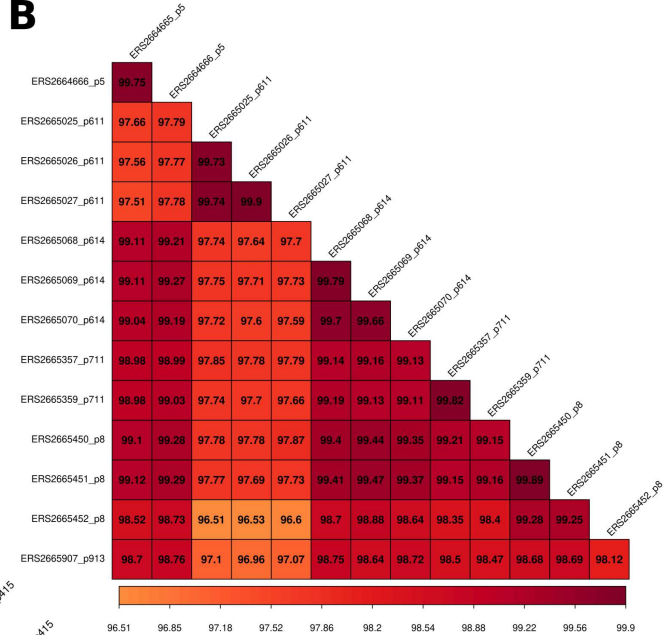

Supplement: FIG S2 [file mbio.03456-22-s0002.pdf]

**A**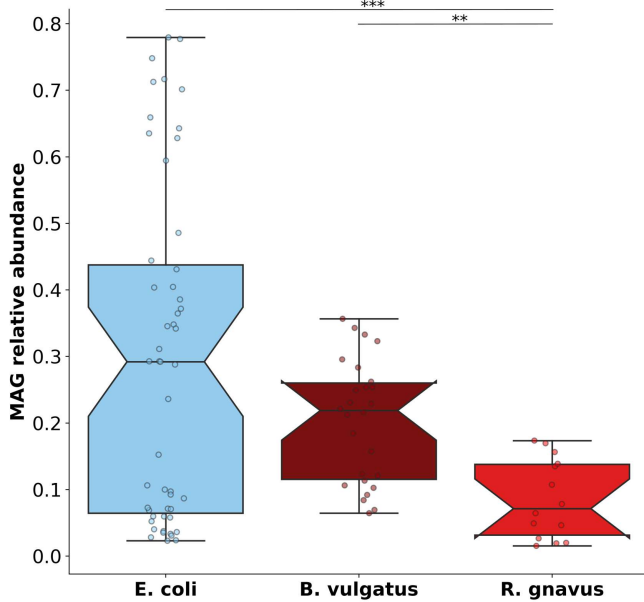**B**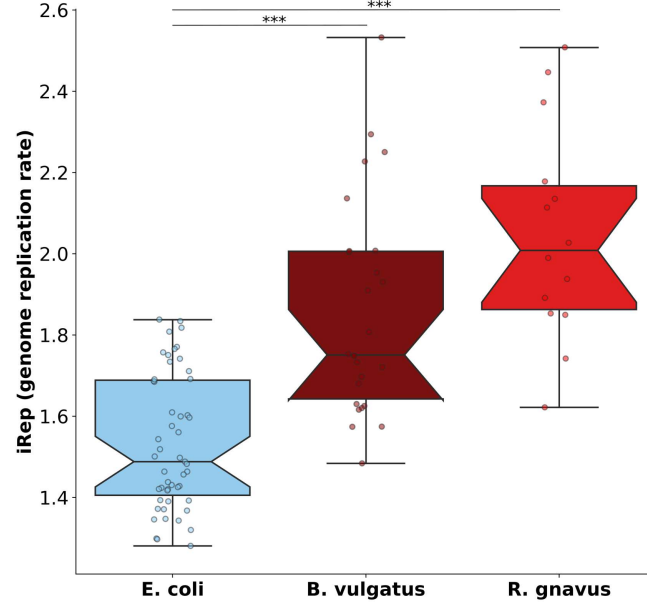**C**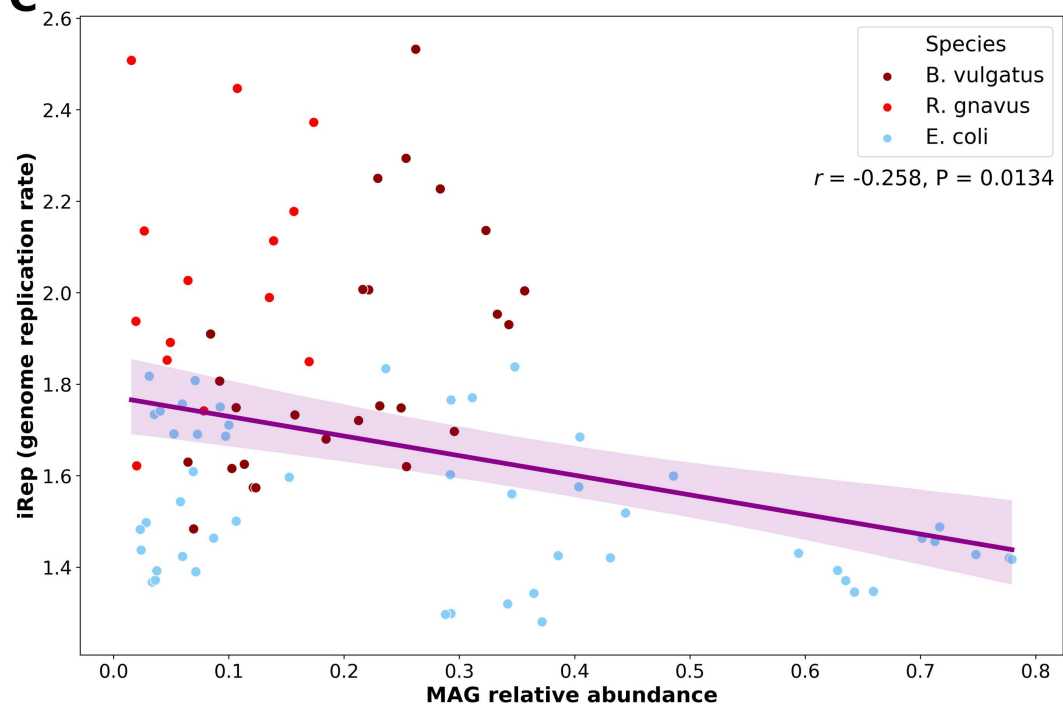

Supplement: FIG S3 [file mbio.03456-22-s0003.pdf]

**A**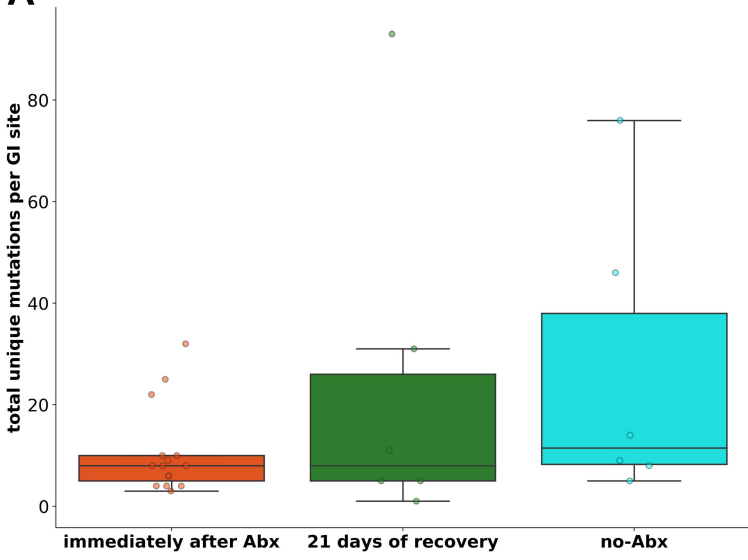**B**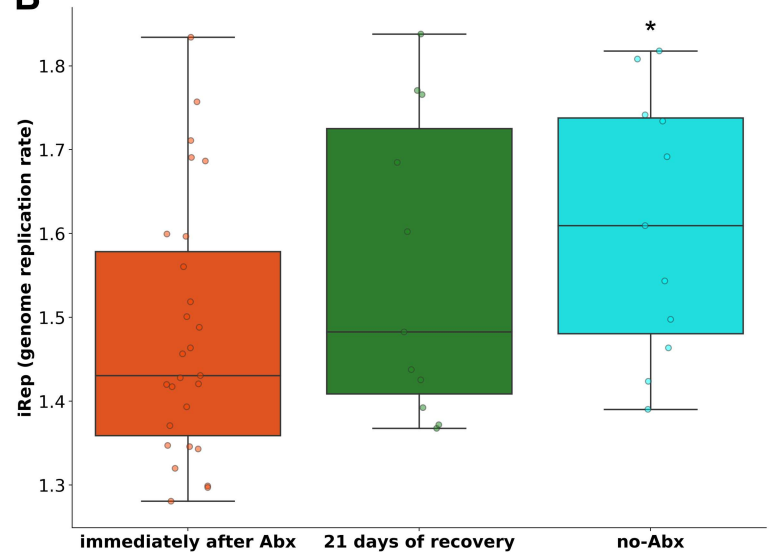

Supplement: FIG S5 [file mbio.03456-22-s0005.pdf]
