# Supplementary material for: Strains Colonizing Different Intestinal Sites within an Individual Are Derived from a Single Founder Population
Source: mBio. 2023 Jan 31;14(1):e03456-22. doi: 10.1128/mbio.03456-22 (PMC9972980; doi:10.1128/mbio.03456-22)
Supplement: FIG S4 [file mbio.03456-22-s0004.pdf]

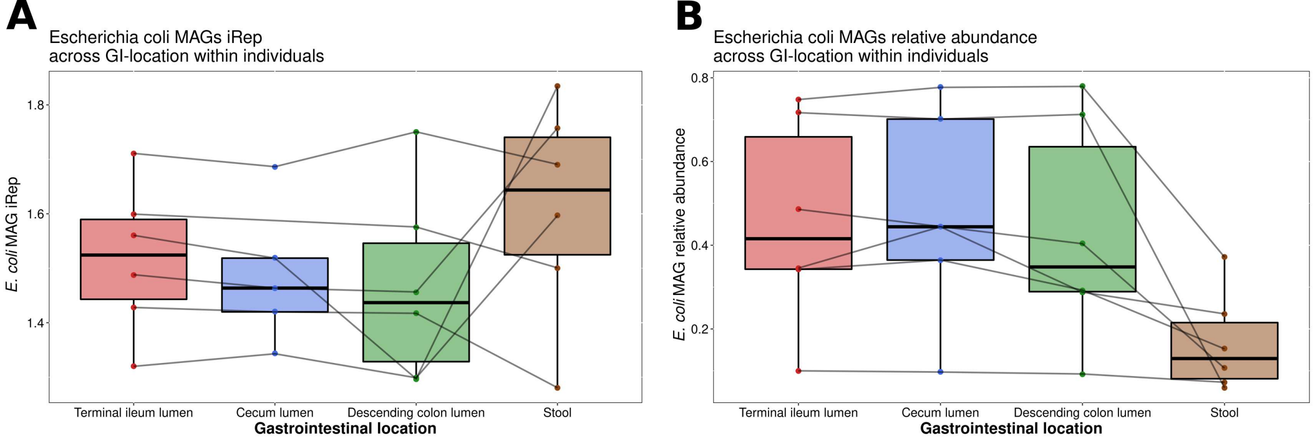
A
Escherichia coli MAGs iRep
across GI-location within individuals
E. coli MAG iRep
1.8
1.6
1.4
Terminal ileum lumen
Cecum lumen
Descending colon lumen
Stool
Gastrointestinal location
B
Escherichia coli MAGs relative abundance
across GI-location within individuals
E. coli MAG relative abundance
0.8
0.6
0.4
0.2
Terminal ileum lumen
Cecum lumen
Descending colon lumen
Stool
Gastrointestinal location
